# Новый способ дифференциальной диагностики гипогонадотропного гипогонадизма и конституциональной задержки полового развития у юношей 13,5–17 лет

**DOI:** 10.14341/probl13419

**Published:** 2024-03-09

**Authors:** Ю. Л. Скородок, И. Ю. Иоффе, Е. В. Плотникова, И. И. Нагорная, Л. А. Желенина, А. В. Кожевникова

**Affiliations:** Санкт-Петербургский государственный педиатрический медицинский университет; Санкт-Петербургский государственный педиатрический медицинский университет; Санкт-Петербургский государственный педиатрический медицинский университет; Санкт-Петербургский государственный педиатрический медицинский университет; Санкт-Петербургский государственный педиатрический медицинский университет; Санкт-Петербургский государственный педиатрический медицинский университет

**Keywords:** гипогонадотропный гипогонадизм, юноши, синдром задержки пубертата, дифференциальная диагностика

## Abstract

ОБОСНОВАНИЕ. Дифференциальная диагностика гипогонадотропного гипогонадизма (ГГ) и конституциональной задержки полового развития (КЗПР) крайне важна, так как при последней половое созревание начинается и полностью завершается без какого-либо медицинского вмешательства, а при первом пубертатный период не наступает либо проходит не до конца, и отсутствие своевременного лечения приводит к медицинской и психосоциальной дезадаптации.ЦЕЛЬ. Разработка метода дифференциальной диагностики гипогонадотропного гипогонадизма и конституциональной задержки полового развития у юношей 13,5–17 лет путем балльной оценки уровней лютеинизирующего гормона, фолликулостимулирующего гормона, тестостерона и ингибина В.МАТЕРИАЛЫ И МЕТОДЫ. Целевую популяцию формировали из юношей 13,5–17 лет с синдромом задержки пубертата путем сплошного включения наблюдений. Оценивали анамнестические данные, стадию пубертата, объем тестикул; определяли сывороточные уровни ЛГ, ФСГ, тестостерона (Т) методом хемилюминисцентного анализа, ингибина В, АМГ–ИФА. Проводили стимуляционные тесты с трипторелином и 3-дневным введением хорионического гонадотропина. Пациентов наблюдали амбулаторно в течение 6–24 месяцев.РЕЗУЛЬТАТЫ. В исследование включили юношей 13,5–17 лет с синдромом задержки пубертата: 56 — с целью создания метода дифференциальной диагностики, 30 — его контроля (группа контроля). Разработали количественный способ, позволяющий дифференцировать ГГ и КЗПР. На основании проведенного ROC-анализа установили наиболее чувствительные и специфичные маркеры ГГ и выбрали из них доступные для амбулаторного исследования базальные уровни ЛГ, ФСГ, Т, ингибина В. Для каждого показателя, основываясь на результатах собственного исследования и данных литературы, подобрали интервалы значений, в зависимости от них — оценки ЛГ, ФСГ, Т и ингибина В, а также коэффициенты (k) для каждого показателя. Баллы вычисляли путем умножения оценки на k, после чего суммировали и нормализовали к максимальной сумме, которую мог получить пациент. Для повышения точности диагностики ввели возрастной коэффициент. Итогом вычисления стал результат балльной оценки (S). S при КЗПР (10,65 [3,13–14,91]) статистически значимо отличался от такового при ГГ (76,46 [57,79–83,74]) (p< 0,001). Поставленные на основании S (<21,16 и ≥55,07) диагнозы в группе контроля подтверждены данными катамнеза в 97% случаев. Разработан алгоритм дифференциальной диагностики ГГ и КЗПР с использованием S.ЗАКЛЮЧЕНИЕ. Результат балльной оценки уровней ЛГ, ФСГ, тестостерона, ингибина В ≥55,07 позволяет диагностировать гипогонадотропный гипогонадизм, < 21,16 — конституциональную задержку полового развития с высокой вероятностью. В случае суммы баллов ≥21,16, но < 55,07 требуется расчет отношения ингибин В/АМГ и/или проведение стимуляционных тестов.

## Abstract (en)

BACKGROUND: Differential diagnosis of hypogonadotropic hypogonadism (HH) and constitutional delay of puberty (CDP) is extremely important since with the latter puberty begins and completes without any medical intervention and in the case of HH puberty does not occur or is incomplete. Failure to start treatment on time leads to medical and psychosocial maladjustment of the patient.AIM: Development of a method for differential diagnosis of hypogonadotropic hypogonadism and constitutional delay of puberty in boys 13.5–17 years old by scoring the levels of LH, FSH, testosterone and inhibin B.MATERIALS AND METHODS: The study group was formed by adolescent men 13.5–17 years old with delayed puberty including all observations. Anamnesis, stage of puberty, testicular volume were assessed; serum levels of LH, FSH, testosterone (T) were determined by chemiluminescent analysis and inhibin B, AMH by ELISA. Stimulation tests were performed with triptorelin and human chorionic gonadotropin (3 days). Patients were followed up for 6–24 months.RESULTS: The study included adolescent men at the age of 13.5–17 years with delayed puberty: 56 for the purpose of development a method of differential diagnosis, 30 for its control (control group). We`ve created a method that allows differentiate HH and CDP. Through the ROC-analysis the most sensitive and specific HH markers were identified. The basal levels of LH, FSH, T, and inhibin B were selected as most available for outpatient testing. Based on the results of our own research and scientific data we selected ranges of values and rated LH, FSH, T and inhibin B depending on them (marks). Then we assigned the coefficients (k) for each hormone. Scores were calculated by multiplying the marks by k then summed and normalized to the maximum amount the patient could get. To increase the accuracy of diagnosis an age coefficient was introduced. The result of the calculation was the result of the scoring (S). S for CDP (10.65 [3.13–14.91]) differed significantly from that for HH (76.46 [57.79–83.74]) (p< 0.001). Diagnoses based on S (<21.16 and ≥55.07) in the control group were confirmed by follow up data in 97% cases. An algorithm for the differential diagnosis of HH and CDP by using S has been developed.CONCLUSION: The result of scoring of LH, FSH, testosterone, inhibin B levels ≥55.07 makes it possible to diagnose hypogonadotropic hypogonadism, < 21.16 — constitutional delay of puberty with a high probability. In the case of score ≥21.16 but < 55.07, calculation of the inhibin B/AMH ratio and/or stimulation tests are required.

## ОБОСНОВАНИЕ

Синдром задержки пубертата у юношей — отставание сроков полового созревания более чем на 2 стандартных отклонения в сравнении с популяцией (традиционно, позже 13,5 лет, причинами которого могут быть стойкие нарушения гипоталамо-гипофизарной и/или гонадной функций (гипогонадизм) и транзиторная недостаточность гонадотропин-рилизинг гормона (задержка полового развития, чаще — конституциональная задержка полового развития, КЗПР) [1][2]. По уровню поражения в системе гипоталамус-гипофиз-гонады выделяют гипогонадотропный (ГГ) и гипергонадотропный гипогонадизм; диагностика последнего не вызывает затруднений в связи с наличием высокоспецифичного и чувствительного биохимического маркера — гипергонадотропинемии [3][4]. В противоположность этому отличить ГГ от КЗПР не всегда просто, так как стойкая или временная гонадотропная недостаточность при этих состояниях имеет сходные клиническую картину и биохимические маркеры [2][3][4]. Однако дифференциальная диагностика этих состояний крайне важна, так как при КЗПР половое созревание начинается и полностью завершается без какого-либо медицинского вмешательства, а при ГГ пубертатный период не наступает либо проходит не до конца [3][5]. Отсутствие своевременного лечения при ГГ приводит к развитию медицинской и психосоциальной дезадаптации [6][7][8].

Ряд исследователей предлагает основываться на базальных уровнях лютеинизирующего гормона (ЛГ), фолликулостимулирующего гормона (ФСГ), тестостерона (Т), ингибина В и антимюллерова гормона (АМГ), однако пороговые значения и выбор показателей в разных публикациях различаются [4][9][10]. Указанные авторы считают, что значения всех показателей у пациента должны быть ниже или выше диагностических уровней для ГГ или КЗПР соответственно, и это является недостатком, потому что при несоблюдении данного условия в отношении хотя бы одного показателя дифференциальная диагностика становится затруднительной.

С другой стороны, отсутствуют стандартные рекомендации по выбору, проведению и оценке стимуляционных тестов [1][4][11]; некоторые авторы считают их вообще неинформативными [12][13].

## ЦЕЛЬ ИССЛЕДОВАНИЯ

Разработка метода дифференциальной диагностики гипогонадотропного гипогонадизма и конституциональной задержки полового развития у юношей 13,5–17 лет путем балльной оценки уровней ЛГ, ФСГ, тестостерона и ингибина В.

## МАТЕРИАЛЫ И МЕТОДЫ

## Место и время проведения исследования

Место проведения. Федеральное государственное бюджетное учреждение высшего профессионального образования «Санкт-Петербургский государственный педиатрический медицинский университет» Минздрава России, Санкт-Петербург, Россия.

Время исследования. Май 2017 года — март 2023 года.

## Изучаемые популяции (одна или несколько)

Популяция: «Синдром задержки пубертата».

Критерии включения: мужской пол, возраст 13,5–17 лет, несоответствие стадии полового развития по Tanner возрасту (I или II у подростков старше 15 лет), согласие на проведение обследования и обработку персональных данных.

Критерии исключения: неконтролируемые соматические или другие эндокринные заболевания и/или нестабильная заместительная терапия, гипергонадотропинемия, численные и структурные перестройки хромосом, синдромы с множественными врожденными пороками развития.

## Способ формирования выборки из изучаемой популяции (или нескольких выборок из нескольких изучаемых популяций)

Путем сплошного включения наблюдений.

## Дизайн исследования

Одноцентровое, обсервационное, с применением диагностических медицинских вмешательств в интересах исследования, динамическое, ретроспективное (6–24 месяца), одновыборочное, контролируемое, сравнительное, не рандомизированное, не маскированное.

## Методы

Критерии включения определяли методом оценки стадии пубертата по Tanner, подписанием информированного добровольного согласия на обследование; исключения — изучения анамнестической медицинской документации, анализа результатов исследования ТТГ, свТ4, кортизола, инсулиноподобного фактора роста-1, ЛГ, ФСГ.

Собирали анамнез заболевания и жизни, проводили анализ сопутствующих состояний, перенесенных заболеваний, хирургических и иных вмешательств. Стадию полового созревания оценивали по Tanner, объем тестикул — орхидометром Prader. Определяли уровни гормонов в сыворотке крови: ЛГ, ФСГ, Т с использованием иммунохемилюминисцентного метода (Cobas, Roche Diagnostics), ингибина В, АМГ — методом иммуноферментного анализа (DSL).

Проводили стимуляционный тест с трипторелином: определяли уровни ЛГ до и через 60, 240 минут после подкожного введения 0,1 мг трипторелина. Пик секреции ЛГ>10 МЕ/л расценивали как положительный тест, ≤10 МЕ/л — отрицательный [1][3]. Проводили 3-дневный стимуляционный тест с хорионическим гонадотропином (ХГ), или функциональную пробу тестикул (ФПТ3): определяли уровень Т до и через 24 часа после трехкратного ежедневного внутримышечного введения ХГ в дозе 1500 МЕ/м². Положительным результатом считали повышение уровня Т>3,5 нмоль/л, отрицательным — ≤3,5 нмоль/л [1][3][4].

Пациентов наблюдали амбулаторно в течение 6–24 месяцев, обследовали повторно/неоднократно при необходимости, части пациентов проводили пробную терапию препаратами Т в дозе 50–100 мг 1 раз в 28 дней внутримышечно в течение 3 месяцев. Спонтанное или индуцированное пробной терапией прогрессирование пубертата расценивали как КЗПР.

## Статистический анализ

Статистическую обработку данных проводили с использованием программного пакета Microsoft Office Excel и StatTech v. 2.8.8 (разработчик ООО «Статтех», Россия). Количественные показатели оценивали на предмет соответствия нормальному распределению с помощью критерия Шапиро-Уилка. Количественные показатели, имеющие нормальное распределение, описывали с помощью средних арифметических величин (M) и стандартных отклонений (SD), границ 95% доверительного интервала. В случае отсутствия нормального распределения количественные данные описывали с помощью медианы (Me) и нижнего и верхнего квартилей [Q1–Q3]. Сравнение групп по количественному показателю, имеющему нормальное распределение, выполняли с помощью t-критерия Уэлча. Сравнение двух групп по количественному показателю, распределение которого отличалось от нормального, выполняли с помощью U-критерия Манна-Уитни. Для оценки диагностической значимости количественных признаков при прогнозировании определенного исхода применяли метод ROC-анализа. Разделяющую величину количественного признака в точке cut-off определяли по наивысшему значению индекса Юдена.

## Этическая экспертиза

Исследование выполнено в рамках диссертационной работы, одобренной юридическим отделом и этическим комитетом Федерального государственного бюджетного образовательного учреждения высшего образования «Санкт-Петербургский государственный педиатрический медицинский университет» Минздрава России (протокол № 5/8 от 18 мая 2017 г.).

## РЕЗУЛЬТАТЫ

В исследование включили юношей 13,5–17 лет (14,50±1,14 (14,17–14,85)) с синдромом задержки пубертата: 56 — с целью создания метода дифференциальной диагностики, 30 — контроля разработанного метода.

Из 56 обследованных у 13 (23,2%) была множественная недостаточность гормонов аденогипофиза (МНГА) (дефицит гормона роста и ТТГ у всех, дефицит АКТГ — у 7, несахарный диабет — у 6), 43 (76,8%) не имели истории другой эндокринной или соматической патологии – изолированная задержка пубертата (ИЗП). У пациентов с МНГА суммарный объем тестикул составил 3,5 [ 2,75–4,0] мл и соответствовал допубертатному возрасту. У большинства пациентов (12; 93,3%) лобковое оволосение отсутствовало, у 1 (6,7%) соответствовало II стадии по Tanner. Медианные уровни ЛГ 0,1 [ 0,07–0,2] МЕ/л и Т 0,04 [ 0–0,21] нмоль/л, средние концентрации ФСГ 0,42±0,31 (0,22–0,62) МЕ/л, были ниже референтного интервала по возрасту и соответствовали допубертатным значениям [14]. Медианные уровни АМГ 25,94 [ 22,27–35,26] нг/мл были выше, а ингибина В 29,05 [ 16,5–47,5] пг/мл — ниже средневозрастных показателей и также соответствовали допубертатным значениям [15][16]. Таким образом, у пациентов пубертатного возраста с МНГА в условиях компенсированного дефицита гормонов гипофиза такие клинические признаки, как допубертатные размеры семенников, отсутствие или недостаточное развитие лобкового оволосения, а также лабораторные данные (не соответствующие возрасту уровни гонадотропных гормонов, Т, ингибина В, АМГ) позволили расценить вероятность ГГ как высокую.

У 43 пациентов с ИЗП суммарный объем яичек составил 6,0 [ 3,5–7,0] мл и соответствовал допубертатному возрасту. Более чем у половины пациентов лобковое оволосение отсутствовало (28; 65,1%), у остальных соответствовало II (14; 32,6%) или III (1; 2,3%) стадии по Tanner. Костный возраст у юношей с ИЗП составил 12,7±1,5 года. 5 пациентов с ИЗП в возрасте от 15 лет 3 мес до 17 лет 6 мес имели крайне низкие уровни ЛГ (0–0,3 МЕ/л), ФСГ (0–0,99 МЕ/л), Т (0,14–0,4 нмоль/л) и ингибина В (16–23 пг/мл). Клинически у этих пациентов не было признаков пубертата, что наряду с лабораторными данными делало высоко вероятным диагноз ГГ. Из оставшихся 38 пациентов другие 10 (возраст 15–15,5 лет) имели не менее двух лабораторных и одного клинического признаков начала пубертата: пубертатные уровни ЛГ (1,01–4,5 МЕ/л) в сочетании с ФСГ (1,27–5,3 МЕ/л) и/или Т (0,18–6,12 нмоль/л) и/или суммарный объем тестикул 9–11 мл. Это делало высоко вероятным диагноз КЗПР. Оставшихся 28 пациентов из группы ИЗП мы отнесли к так называемой «серой зоне», когда ГГ не был исключен, и сохранялась вероятность КЗПР. Медианный уровень ЛГ 0,38 [ 0,11–1,79] МЕ/л соответствовал нижнему квартилю нормального пубертатного интервала, тогда как среднее содержание ФСГ 1,75±1,23 (1,26–2,24) МЕ/л, а также медианные концентрации Т 0,545 [ 0,15–1,04] нмоль/л и ингибина В 35,00 [ 19,0–112,10] пг/мл были ниже, а средний уровень АМГ 31,56±21,98 (15,84–47,28) нг/мл — выше пубертатных значений [14][15][16]. При этом концентрации ЛГ, ФСГ и Т были статистически значимо выше, чем у пациентов группы МНГА (р=0,027, <0,001 и 0,006 соответственно).

Результаты ROC-анализа различающихся показателей представлены в таблице 1.

**Table:** Таблица 1. Результаты ROC-анализа уровней ЛГ, ФСГ и тестостерона у пациентов с гипогонадотропным гипогонадизмом в структуре множественной недостаточности гормонов аденогипофиза и изолированной задержкой пубертата * — уровень, которому соответствует наивысшее значение индекса Юдена. ГГ прогнозировался при значениях ниже данной величины или равном ей;** — статистическая значимость модели.

Показатель	Пороговый уровень*	Se, %	Sp, %	Площадь под ROC-кривой	p**
ЛГ, МЕ/л	0,23	83,3	69,8	0,731±0,084 (0,565–0,896)	0,027
ФСГ, МЕ/л	0,9	100	81,4	0,837±0,065 (0,709–0,964)	0,001
Тестостерон, нмоль/л	0,11	75,0	87,8	0,788±0,075 (0,641–0,935)	0,006

Пациентам из «серой зоны» ИЗП провели тест с трипторелином: положительный результат теста отмечался у 10 юношей, отрицательный — у 18.

13 пациентов с МНГА, 5 с крайне низкими уровнями ЛГ, ФСГ, Т, ингибина В, а также 12 (из 18 с отрицательным тестом с трипторелином) без признаков прогрессирования полового развития, по данным катамнеза, составили подгруппу пациентов с диагностированным ГГ (подгруппа 1, 30 юношей). По результатам катамнеза, 6 пациентов из 18 с отрицательным тестом демонстрировали прогрессирование самостоятельного или индуцированного препаратами Т пубертата, что позволило диагностировать у них КЗПР (подгруппа 2). Пациенты подгрупп 1 и 2 были сопоставимы по возрасту (р=0,613). Уровни ЛГ, ФСГ, Т и ингибина В у пациентов обеих подгрупп представлены в таблице 2.

**Table:** Таблица 2. Уровни ЛГ, ФСГ, тестостерона и ингибина В у пациентов подгрупп 1 и 2 * — различия показателей статистически значимы (p<0,05).

Показатель Me [ Q1-Q3]/M ± SD (95% ДИ)	Подгруппа	р	Норма для II–IV стадии пубертата по Tanner [14][16]
1 (n=30)	2 (n=6)
ЛГ, МЕ/л	0,1 [ 0,05–0,29]	0,48 [ 0,18–0,68]	0,107	0,2–7,0
ФСГ, МЕ/л	0,64 [ 0,19–0,94]	2,51 [ 2,04–2,74]	<0,001*	1,8–9,2
Тестостерон, нмоль/л	0,17 [ 0,01–0,48]	0,22 [ 0,11–0,48]	р=0,86	0,62–21,5
Ингибин В, пг/мл	21 [ 15,25–42,12]	35 [ 34,5–73,55]	р=0,115	62–338

Как видно из таблицы 2, концентрация ФСГ была статистически значимо выше у пациентов подгруппы 2. Различий между уровнями ЛГ, Т, ингибина В у пациентов подгрупп 1 и 2 не было. По результатам ROC-анализа, пороговый уровень ФСГ в точке cut-off, которому соответствовало наивысшее значение индекса Юдена, составил 1,58 МЕ/л. ГГ прогнозировался при значениях ниже данной величины или равном ей (чувствительность 100% и специфичность 89,7%). Площадь под ROC-кривой составила 0,976±0,045 (0,888–1,000); модель была статистически значимой (p<0,001).

В заключение провели сравнение групп пациентов с диагностированным ГГ (30 пациентов) и КЗПР (26 пациентов). Группу КЗПР составили 10 пациентов с клиническими и/или лабораторными признаками начала пубертата, 10 пациентов с положительным результатом теста с трипторелином и 6 пациентов с отрицательным результатом теста с трипторелином, но прогрессирующим в динамике пубертатом. Пациенты групп ГГ и КЗПР были сопоставимы по возрасту (р=0,089). Суммарный объем тестикул у пациентов с ГГ был статистически значимо меньше, чем при КЗПР: 3,94±2,51 (2,49–5,39) и 6,2±3,08 (4,98–7,42) мл соответственно (р=0,023). Базальные и стимулированные уровни ЛГ и Т, концентрации ФСГ, ингибина В, АМГ и отношение ингибин В/АМГ у пациентов с ГГ и КЗПР представлены в таблице 3.

**Table:** Таблица 3. Базальные и стимулированные уровни ЛГ и тестостерона, концентрации ФСГ, ингибина В, АМГ и отношение ингибин В/АМГ у пациентов с гипогонадотропным гипогонадизмом и конституциональной задержкой полового развития * — различия показателей статистически значимы (p<0,05); Т (ФПТ3) — тестостерон, стимулированный трехкратным введением хорионического гонадотропина; ГГ — гипогонадотропный гипогонадизм; КЗПР — конституциональная задержка полового развития.

Показатель	Диагноз	P
ГГ (n=30) Me [ Q1–Q3] M±SD (95% ДИ)	КЗПР (n=26) Me [ Q1–Q3] M±SD (95% ДИ)
ЛГ, МЕ/л	0,1 [ 0,01–0,28]	1,04 [ 0,54–2,04]	<0,001*
ФСГ, МЕ/л	0,69 [ 0,2–0,96]	2,55 [ 1,75–3,2]	<0,001*
Тестостерон, нмоль/л	0,2 [ 0,01–0,45]	1,0 [ 0,48–1,6]	<0,001*
Стимулированный ЛГ, МЕ/л	1,09 [ 0,3–4,04]	13,1 [ 9,66–20,24]	<0,001*
Т (ФПТ3), нмоль/л	2,14 [ 1,61–4,22] N=12	10,1 [ 6,69–13,25] N=14	0,005*
Ингибин В, пг/мл	23 [ 16,0–46,0]	112,1 [ 47,95–146,15]	0,013*
Антимюллеров гормон, нг/мл	26,06±11,81 (18,55–33,56)	55,78±6,03 (46,19–65,38)	<0,001*
Отношение ингибин В/АМГ	0,97 [ 0,66–1,68]	2,60 [ 2,32–2,84]	0,039*

Как видно из таблицы 3, все исследованные показатели достоверно различались у пациентов групп ГГ и КЗПР.

Результаты ROC-анализа различающихся показателей представлены в таблице 4.

**Table:** Таблица 4. Результаты ROC-анализа базальных и стимулированных уровней ЛГ и тестостерона, концентраций ФСГ, ингибина В, АМГ и отношения ингибин В/АМГ у пациентов с гипогонадотропным гипогонадизмом и конституциональной задержкой полового развития * — уровень, которому соответствует наивысшее значение индекса Юдена. ГГ прогнозировался при значениях ниже данной величины или равном ей;** — статистическая значимость модели;Т (ФПТ3) — тестостерон, стимулированный трехкратным введением хорионического гонадотропина.

Показатель	Пороговый уровень*	Se, %	Sp, %	Площадь под ROC-кривой	p**
ЛГ, МЕ/л	0,38	82,8	85,2	0,916±0,040 (0,836–0,995)	<0,001
ФСГ, МЕ/л	1,27	86,2	92,6	0,951±0,031 (0,891–1,000)	<0,001
Тестостерон, нмоль/л	0,48	75,9	76	0,826±0,059 (0,711–0,941)	<0,001
Стимулированный ЛГ, МЕ/л	8,62	95,0	88,2	0,969±0,031 (0,909–1,000)	<0,001
Ингибин В, пг/мл	35	66,7	100,0	0,929±0,072 (0,787–1,000)	0,002
Антимюллеров гормон, нг/мл	50,25	91,7	100,0	0,962±0,071 (0,823–1,000)	0,007
Ингибин В/АМГ	1,872	83,3	100,0	0,939±0,101 (0,741–1,000)	0,024
Т (ФПТ3), нмоль/л	5,84	85,7	80,0	0,860±0,073 (0,716–1,000)	0,001

Для создания количественного способа дифференциальной диагностики ГГ и КЗПР установили наиболее чувствительные и специфичные маркеры ГГ и выбрали из них доступные в амбулаторных условиях уровни ЛГ, ФСГ, Т, ингибина В. Для каждого показателя, основываясь на результатах собственного исследования и данных литературы, подобрали интервалы значений и начисляемые баллы. Критические точки уровней гормонов представлены в таблице 5.

**Table:** Таблица 5. Критические точки уровней ЛГ, ФСГ, тестостерона, ингибина В * при сравнении пациентов с множественной недостаточностью гормонов аденогипофиза и изолированной задержкой пубертата;** при сравнении пациентов с гипогонадотропным гипогонадизмом и конституциональной задержкой полового развития;*** нижний квартиль нормы II стадии по Tanner [14][16];**** при сравнении пациентов с гипогонадотропным гипогонадизмом и конституциональной задержкой полового развития с отрицательным тестом с трипторелином.

Показатель	Критическая точка	Чувствительность, %	Специфичность, %
ЛГ, МЕ/л	0,23*	83,3	69,8
0,38**	82,8	85,2
1,24***		
ФСГ, МЕ/л	0,9*	100,0	81,4
1,27**	86,2	92,6
1,58****	100	89,7
Тестостерон, нмоль/л	0,11*	75,0	87,8
0,48**	75,9	76
1,7***		
Ингибин В, пг/мл	35**	66,7	100
65***		

Эмпирическим путем подобрали оценки ЛГ, ФСГ, Т и ингибина В в зависимости от диапазона показателей (таблица 6).

**Table:** Таблица 6. Расчет оценки ЛГ, ФСГ, тестостерона и ингибина В в зависимости от диапазона соответствующих показателей Т — тестостерон.

Показатель	Диапазон показателей	Оценка
ЛГ, МЕ/л	≤0,23	-13,04×ЛГ+5
>0,23, но ≤0,38	-6,67×ЛГ+3,53
>0,38, но ≤1,24	-1,16×ЛГ+1,44
>1,24	0
ФСГ, МЕ/л	≤0,9	-3,33×ФСГ +6
>0,9, но ≤1,27	-2,27×ФСГ +5,43
>1,27, но ≤1,58	-6,45×ФСГ + 10,19
>1,58	0
Т, нмоль/л	≤0,11	-27,27×Т +6
>0,11, но ≤1,48	-2,7×Т +3,3
>0,48, но ≤1,7	-1,64×Т +2,79
>1,7	0
Ингибин В, пг/мл	≤35	-0,07×Ингибин В+10
>35, но ≤65	-0,25×Ингибин В+16,25
>65	0

С учетом чувствительности показателей подобрали коэффициенты (k): для ЛГ, Т и ингибина В постоянные — 8,3, 7,5 и 6 соответственно. Для ФСГ, в связи с разной чувствительностью, для разных групп в исследовании коэффициент варьировал: при значениях 0,9<ФСГ≤1,58 МЕ/л коэффициент 8,6, ≤0,9 или >1,58 МЕ/л — 10.

Баллы за каждый показатель вычисляли путем умножения оценки (таблица 6) на k, после чего баллы суммировали. В связи с ограниченной доступностью исследования ингибина В сумму баллов нормализовали к максимальной сумме (Max), которую мог получить пациент. Результат нормализации выражали в процентах.

 (1)

где ND — normalized data (нормализованная сумма баллов)

Баллы ЛГ = Оценка ЛГ×kЛГ (2)

Баллы ФСГ = Оценка ФСГ×kФСГ (3)

Баллы Т = Оценка Т×kT (4)

Баллы Ингибин В = Оценка Ингибин В×kИнгибин В (5)

Max = maxЛГ+maxФСГ+maxТ+maxИ (6)

 (7)

 (8)

 (9)

 (10)

Дальнейший анализ разработанного метода показал, что точность диагностики ГГ можно повысить, используя поправку на возраст пациента, поскольку диапазон 13,5–17 лет является слишком широким и включает все стадии пубертата у здоровых юношей. В связи с этим в расчет ND ввели возрастной коэффициент (аge, A), полученный эмпирическим путем.

При возрасте ≤15 лет, A=1; >15÷<18 лет, чтобы добиться непрерывного увеличения A, использовали метод кубической апроксимации:

 (11)

где y (years) — возраст в годах

Балльную оценку (score, S) рассчитывали по формуле:

 (12)

Таким образом, с увеличением возраста пациента отмечается возрастание S, что делает более специфичной диагностику ГГ у пациентов старшего возраста с сомнительными результатами ND.

Распределение S у пациентов исследования в зависимости от диагноза представлено на рисунке 1.

**Figure:**
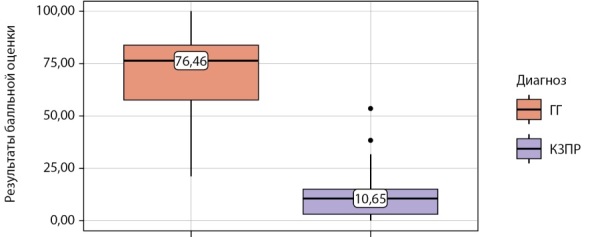
Рисунок 1. Результат балльной оценки у пациентов с гипогонадотропным гипогонадизмом и конституциональной задержкой полового развития.

Как видно из рисунка 1, медианные значения S при КЗПР составили 10,65 [ 3,13–14,91], при ГГ — 76,46 [ 57,79–83,74] (p<0,001).

Пороговые значения S и результаты ее ROC-анализа представлены в таблице 7 и на рисунках 2, 3.

**Table:** Таблица 7. Пороговые значения результата балльной оценки PPV (positive predictive value) — прогнозируемая ценность положительного результата.NPV (negative predictive value) — прогнозируемая ценность отрицательного результата.

Порог	Чувствительность, %	Специфичность, %	PPV	NPV
55,07	78,6	100,0	100,0	81,8
53,51	78,6	96,3	95,7	81,2
40,21	92,9	96,3	96,3	92,9
38,15	92,9	88,9	89,7	92,3
37,98	96,4	88,9	90,0	96,0
27,12	96,4	81,5	84,4	95,7
21,16	100,0	81,5	84,8	100,0

**Figure:**
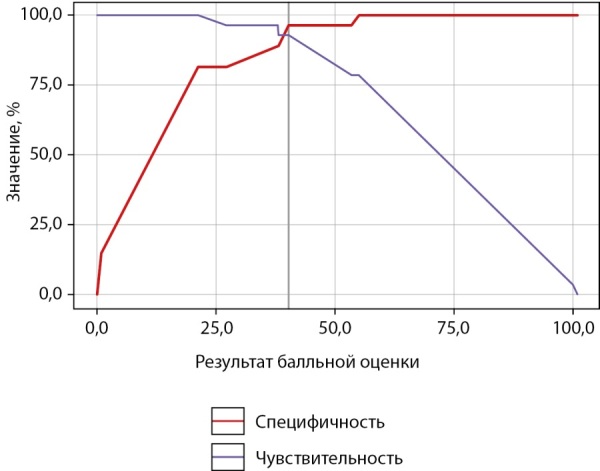
Рисунок 2. Чувствительность и специфичность пороговых значений результата балльной оценки в отношении гипогононадотропного гипогонадизма.

**Figure:**
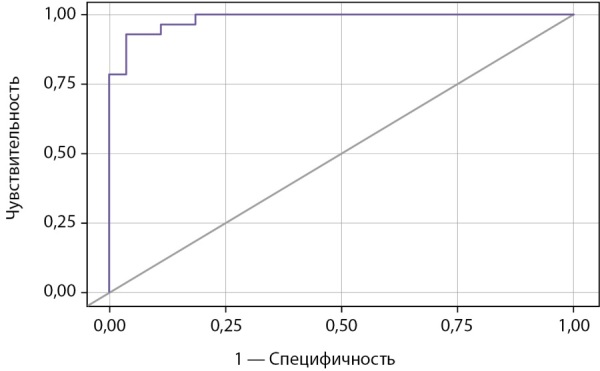
Рисунок 3. ROC-кривая, характеризующая достоверность диагностики гипогононадотропного гипогонадизма на основании результата балльной оценки.

В таблице 7 значения S расположены в порядке увеличения чувствительности и убывания специфичности показателя. Пороговая величина S в точке cut-off, которой соответствовало наивысшее значение индекса Юдена, составила 40,21 (рис. 2). ГГ прогнозировали при значении S выше данной величины или равном ей (чувствительность 92,9% и специфичность 96,3%). Площадь под ROC-кривой (рис. 3) составила 0,984±0,017 (0,950–1,000). Это свидетельствует о том, что установленная пороговая точка S является статистически значимой (p<0,001). Прогностическая ценность метода выражена показателями PPV и NPV: при S<21,16 и ≥55,07 точность положительного и отрицательного (соответственно) прогноза в отношении ГГ составила 100%.

После разработки метода для его контроля обследовали 30 юношей (группа а) 14–17 лет с синдромом задержки пубертата. По результатам катамнеза 21 пациент (70%) демонстрировал прогрессирующий пубертат (подгруппа КЗПРа), у 9 (30%) признаки полового созревания отсутствовали (подгруппа ГГа). S вычисляли по исходным данным пациентов. Последующий анализ выявил статистически значимые различия S между подгруппами КЗПРа 3,56 [ 0,00–22,50] и ГГа 73,86 [ 55,07–78,16] (р<0,001). Поставленные на основании S (<21,16 и ≥55,07) диагнозы подтверждены на основании катамнеза в 97% случаев. Для удобства практического применения разработанного метода создан online калькулятор, доступный по ссылке https://crescente.ioffe.su/. По разработанному методу подана заявка на Евразийский патент №202390831 (МПК А61В 5/02) от 31.03.2023.

Алгоритм дифференциальной диагностики ГГ и КЗПР с использованием S представлен на рисунке 4.

**Figure:**
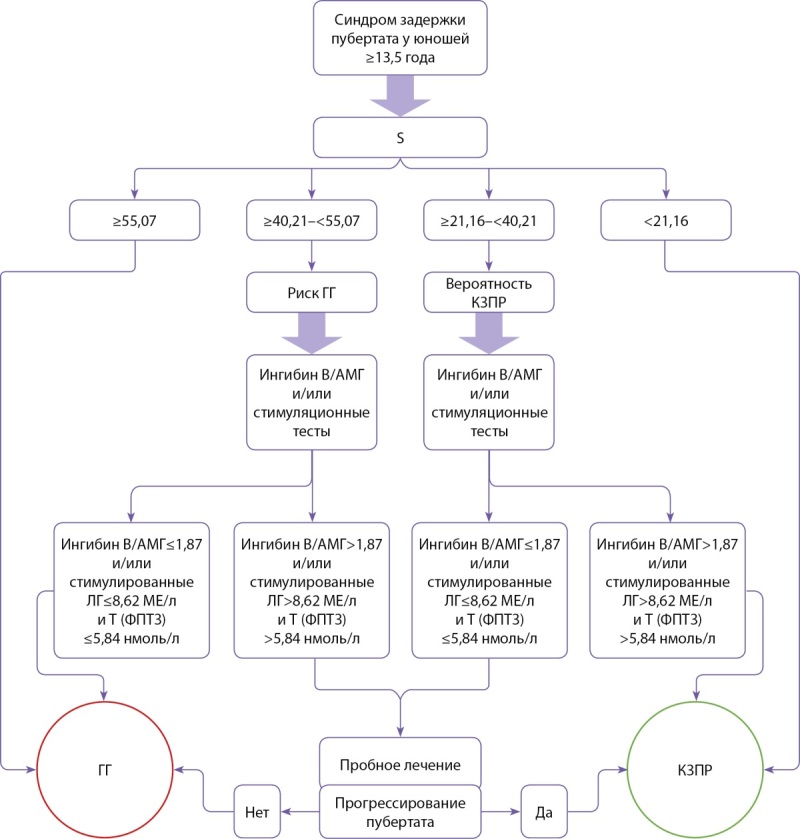
Рисунок 4. Алгоритм дифференциальной диагностики гипогонадотропного гипогонадизма и конституциональной задержки полового развития с использованием балльной оценки (S — результат балльной оценки, Т — тестостерон, Т (ФПТ3) — тестостерон, стимулированный трехкратным введением хорионического гонадотропина, ГГ — гипогонадотропный гипогонадизм, КЗПР — конституциональная задержка полового развития).

## ОБСУЖДЕНИЕ

## Репрезентативность выборок

По данным Росстата, в Санкт-Петербурге (СПб) и Ленинградской области (ЛО) на 01.01.2020 г. проживают 174 032 юноши в возрасте 14–17 лет [17]. Согласно литературным данным, популяционная частота ГГ составляет примерно 1:8000 [1][4]. Таким образом, расчетное количество больных ГГ среди мальчиков-подростков СПб и ЛО — порядка 21 человека, что позволяет считать выборку настоящего исследования репрезентативной.

## Сопоставление с другими публикациями

Возможность использования базальных уровней ЛГ, ФСГ и Т для дифференциальной диагностики ГГ и КЗПР до настоящего времени дискутабельна. По нашим данным, базальные концентрации ЛГ, ФСГ, Т у пациентов с КЗПР были достоверно выше, чем у пациентов с ГГ (р<0,001), ROC-анализ позволил установить наиболее чувствительные и специфичные пороговые уровни этих гормонов в отношении ГГ. Так, пороговый уровень ЛГ в отношении ГГ, по нашим данным, составил ≤0,38 МЕ/л, что не противоречит результатам G. Binder et al. (2014 г.) (<0,3 МЕ/л), A.M. Sequera et al. (2002 г.) (<0,6 МЕ/л) и проекту клинических рекомендаций «Гипогонадизм у детей и подростков» (<0,6 МЕ/л) [4][11][18]. В то же время Латышев О.Ю. и соавт. (2019 г.) указывают на уровень ЛГ>0,3 МЕ/л как признак КЗПР [10].

С целью оптимизации диагностики мы разработали количественный способ, позволяющий дифференцировать ГГ и КЗПР по балльной оценке (S), полученной на основании анализа базальных уровней гормонов: наиболее чувствительных и специфичных, доступных в амбулаторных условиях, имеющих монотонную функцию ЛГ, ФСГ, Т, ингибина В. Оценку каждого показателя проводили на основании полученных в ходе ROC-анализа точек cut-off, их чувствительности, а также литературных данных. Для повышения специфичности метода был введен возрастной коэффициент. В результате, чем ниже были уровни гормонов и чем больше возраст пациента, тем выше оказывалась S, которая у пациентов с ГГ была статистически значимо выше, чем при КЗПР (p<0,001). Проведенный ROC-анализ позволил диагностировать ГГ с 92,9% чувствительностью и 96,3% специфичностью при S≥40,21. Вероятность ГГ при S≥55,07 составляет 100%, <21,16 — 0%; при S≥21,16, но <55,07 может потребоваться дополнительное обследование. Данные о базальном уровне ФСГ как диагностическом критерии ГГ в литературе малочисленны, или не расценивают этот показатель как самостоятельный. По нашим данным, пороговый уровень ФСГ в отношении ГГ составил ≤1,27 МЕ/л (чувствительность 86,2%, специфичность 92,6%) и соответствовал предлагаемому в проекте клинических рекомендаций «Гипогонадизм у детей и подростков», 2021 г. (<1,2 МЕ/л) [4]. Установленный A. M. Sequera et al. (2002 г.) критический в отношении ГГ уровень ФСГ 2,5 МЕ/л имеет меньшую чувствительность (75%) [18]. Данные о базальном уровне Т как диагностическом критерии ГГ в литературе ограничены. По результатам нашего исследования, пороговый уровень Т в отношении ГГ составил ≤0,48 нмоль/л, что значительно ниже значений, предлагаемых Европейским консенсусом и проектом клинических рекомендаций «Гипогонадизм у детей и подростков», 2021 г. (<2 нмоль/л) [4][9]. Пороговый уровень ингибина В в нашем исследовании составил 35 пг/мл, что близко к среднему между соответствующими показателями по данным ряда источников: 28,5 пг/мл (S. Trabado, 2014, J. Rohayem, 2015), 42 пг/мл (M. Hafez et al., 2014), 30 пг/мл (European consensus statement on congenital hypogonadotropic hypogonadism — pathogenesis, diagnosis and treatment, 2015) [9][12][13][19]. Тем не менее, несмотря на 100% специфичность сниженного уровня ингибина В (≤35 пг/мл) в отношении ГГ, недостаточная чувствительность (66,7%) этого показателя не позволяет использовать его для дифференциальной диагностики без оценки других данных. Подобного мнения придерживаются Y. Gao et al. (2021 г.), считающие необходимым оценивать уровень ингибина В в зависимости от степени лобкового оволосения [20], авторы проекта клинических рекомендаций «Гипогонадизм у детей и подростков», 2021 г. — стадии развития наружных половых органов [4]. Некоторые авторы считают возможным использовать ингибин В как критерий ГГ при совместной оценке с другими гормонами [10][11][19].

Аналогичных способов дифференциальной диагностики ГГ и КЗПР в доступной литературе не найдено.

Известные на сегодняшний день методы дифференциальной диагностики ГГ и КЗПР путем оценки базальных уровней ЛГ, ФСГ, Т и, по рекомендациям некоторых авторов, также ингибина В, имеют ряд недостатков: значения всех заявленных показателей у пациента должны быть ниже или выше диагностических уровней для ГГ или КЗПР соответственно, а при несоблюдении данного условия в отношении хотя бы одного показателя дифференциальная диагностика становится затруднительной; разные авторы приводят различные пороговые значения указанных гормонов [4][9][10]. Расчет балльной оценки позволяет нивелировать указанные выше недостатки и проводить дифференциальную диагностику ГГ и КЗПР даже в случае дискордантности полученных результатов или отсутствия одного из них.

Уровни АМГ не включали в расчет суммы баллов, так как функция АМГ в пубертате не монотонна [15]. Принимая во внимание сведения о разнонаправленной динамике уровней ингибина В и АМГ в препубертатном и раннем пубертатном возрасте у здоровых юношей [15], мы использовали ранее не описанное отношение ингибина В к АМГ (ингибин В/АМГ). Целью оценки ингибин В/АМГ было повышение чувствительности и специфичности диагностики. По нашим результатам, подростки с КЗПР имели достоверно более высокие значения ингибин В/АМГ, чем пациенты с ГГ (р=0,039). Установленное нами пороговое значение ингибин В/АМГ≤1,872 обладало достаточной чувствительностью и специфичностью (83,3% и 100% соответственно) в отношении ГГ. Аналогичных результатов в доступной литературе не найдено.

## Клиническая значимость результатов

Разработанный способ позволяет проводить дифференциальную диагностику синдрома задержки пубертата и выявлять на амбулаторном этапе пациентов с ГГ (S ≥55,07), КЗПР (S <21,16) и тех немногих, кому требуется проведение стимуляционных тестов.

## Ограничения исследования

Анализируя факторы, которые могли бы привести к случайным смещениям результатов и, следовательно, повлиять на выводы исследования, считаем необходимым предупредить о возможности ложноотрицательного результата балльной оценки в случаях ГГ, приобретенного после старта пубертата, а также у пациентов с парциальным дефицитом гонадотропинов (ЛГ или ФСГ).

## Направления дальнейших исследований

Дальнейшее изучение особенностей пациентов с синдромом задержки пубертата и формирование более крупных баз данных позволит усовершенствовать предложенные метод и алгоритм дифференциальной диагностики путем коррекции назначенных для показателей диапазонов, оценки и коэффициентов, возрастного коэффициента.

## ЗАКЛЮЧЕНИЕ

Результат балльной оценки уровней ЛГ, ФСГ, тестостерона, ингибина В ≥55,07 позволяет диагностировать гипогонадотропный гипогонадизм, <21,16 — конституциональную задержку полового развития с высокой вероятностью. В случае суммы баллов ≥21,16, но <55,07 требуется расчет отношения ингибин В/АМГ и/или проведение стимуляционных тестов.

## ДОПОЛНИТЕЛЬНАЯ ИНФОРМАЦИЯ

Источники финансирования. Работа выполнена по инициативе авторов без привлечения финансирования, при лабораторно-инструментальном обеспечении Санкт-Петербургского государственного педиатрического медицинского университета.

Конфликт интересов. Авторы декларируют отсутствие явных и потенциальных конфликтов интересов, связанных с содержанием настоящей статьи

Участие авторов. Все авторы одобрили финальную версию статьи перед публикацией, выразили согласие нести ответственность за все аспекты работы, подразумевающую надлежащее изучение и решение вопросов, связанных с точностью или добросовестностью любой части работы.

Благодарности. Авторы благодарят ректора Санкт-Петербургского государственного педиатрического медицинского университета д.м.н., профессора Иванова Дмитрия Олеговича, заведующую эндокринологическим отделением (до 2022 г.) д.м.н., профессора кафедры факультетского педиатрии, заслуженного врача России Тыртову Людмилу Викторовну и заведующую эндокринологическим отделением (с 2022 г.) к.м.н. Скобелеву Кристину Владимировну.
